# The Functional Role of Fecal Microbiota Transplantation on Dextran Sulfate Sodium-Induced Colitis in Mice

**DOI:** 10.3389/fcimb.2019.00393

**Published:** 2019-11-15

**Authors:** Yan He, Xueting Li, Hengyuan Yu, Yixuan Ge, Yuanli Liu, Xiaofa Qin, Mingshan Jiang, Xiuhong Wang

**Affiliations:** ^1^Department of Biochemistry and Molecular Biology, Heilongjiang Provincial Science and Technology Innovation Team in Higher Education Institutes for Infection and Immunity, Harbin Medical University, Harbin, China; ^2^GI Biopharma Inc., Westfield, NJ, United States; ^3^Department of General Surgery, The Second Affiliated Hospital of Harbin Medical University, Harbin, China

**Keywords:** ulcerative colitis, fecal microbiota transplantation, intestinal mucosal barrier, digestive proteases, β-glucuronidase

## Abstract

Increasingly studies revealed that dysbiosis of gut microbiota plays a pivotal role in the pathogenesis of ulcerative colitis (UC). Fecal microbiota transplantation (FMT) has drawn more and more attention and become an important therapeutic approach. This study aims to examine the facts about the effective components and look into potential mechanisms of FMT. Colitis was induced by 3% (w/v) dextran sulfate sodium (DSS) in drinking water for 7 days. Colitis mice were administered by oral gavage with fecal suspension, fecal supernatant, fecal bacteria, or boiling-killed fecal bacteria from healthy controls and the disease activity index was monitored daily. On the seventh day, mice were euthanized. The length, histological score, parameters related to inflammation, gut barrier functions of the colon, activities of digestive protease and β-glucuronidase in feces were measured. All of the four fecal components showed certain degree of efficacy in DSS-induced colitis, while transplantation of fecal suspension showed the most potent effect as demonstrated by less body weight loss, lower disease activity scores, more expression of tight junction proteins and TRAF6 and IκBα, less expression of TNF-α, IL-1β, IL-10, TLR-4, and MyD88 in gut tissue, as well as restoration of fecal β-glucuronidase and decreases in fecal digestive proteases. These results provide a novel insight into the possible mechanism of FMT and may help to improve and optimize clinical use of FMT.

## Introduction

Ulcerative colitis (UC) is one of the main forms of inflammatory bowel disease (IBD) with unknown etiology, mainly characterized as non-specific inflammation of the rectum and colon (Ordás et al., 2012). It emerged for only about a century and has kept increasing worldwide, as shown by the remarkable increase in recent years in Asia (Ng et al., 2015; Bernstein, 2017; Kaplan and Ng, 2017). Although the exact cause of UC remains to be elucidated, evidence from immunological, microbiological and genetic studies suggests that UC results from a deregulated intestinal immune response driven by a complex interplay between the host and intraluminal microbiota (Sartor, 2006). At present, UC is primarily treated with medications targeting inflammation and the immune system, however, the majority of patients eventually become refractory or intolerant over time (Duijvestein et al., 2018; Im et al., 2018; Matsuoka et al., 2018).

More and more studies revealed that dysbiosis of gut microbiota plays pivotal role in the pathogenesis of IBD such as UC (Nishida et al., 2018; Zuo and Ng, 2018). The abundance of intestinal flora in UC patients is significantly reduced, along with changes in composition (Matsuoka and Kanai, 2015). As the result, fecal microbiota transplantation (FMT) for treatment of IBD has become a hot area of extensive research (Imdad et al., 2018; Sunkara et al., 2018). In fact, back to the fourth century, Ge Hong, in the Handbook of Emergency Medicine, prescribed ingestion of feces for a variety of conditions (Faming et al., 2012). Recent study shows that FMT is effective for more than 90% of patients with recurrent *C. difficile* infection (Surawicz et al., 2013). Multiple studies have also demonstrated therapeutic effect of FMT on UC (Costello et al., 2017; D'Odorico et al., 2018), but the specific functional component and mechanism remains to be elucidated.

Study had found that colonic mucosal barrier damage and intestinal flora disorder were observed in the early stage of UC (Rutgeerts et al., 2007; Meerveld, 2012), suggesting that intestinal mucosal barrier function is very important in the pathogenesis of UC. Recently, studies have also shown that regulating intestinal flora can improve intestinal mucosal barrier function (Charlotte et al., 2007; Tran et al., 2015). However, the mechanism is still unclear. It is well-documented that increased gut permeability (“Leaky gut”) has played a critical role in the pathogenesis of IBD (Michielan and D'Inca, 2015; Vindigni et al., 2016), while proteases would be an important damaging factor for gut barrier due to the strong proteolytic action (Biancheri et al., 2013). In fact, Qin demonstrated that digestive proteases can be inactivated by unconjugated but not conjugated bilirubin (Qin, 2007) and proposed that impaired inactivation of digestive proteases by deconjugated bilirubin as the result of the reduction in gut bacteria, thus bacterial β-glucuronidase, along with improved hygiene and inhibition by dietary chemicals such as the widely used artificial sweetener saccharin in modern society may have played a critical role in the pathogenesis of IBD (Qin, 2002). Similarly, our previous studies using bile duct ligation rats have confirmed that unconjugated bilirubin (UCB) inactivated digestive proteases and protected the integrity of the intestinal barrier (Zhou et al., 2014). We further observed that UCB administration ameliorates the tissue damage and inflammation of TNBS-induced colitis accompanied by reduction in fecal trypsin and chymotrypsin (Zhou et al., 2017). However, little attention has been paid to the role of bacterial β-glucuronidase and digestive proteases in the pathogenesis of UC and their relationship with the efficacy of FMT.

In this study, we aim to explore the functional component and possible mechanism of FMT by virtue of administration of dextran sulfate sodium (DSS)-induced colitis mice with different components of fecal material, in hoping to provide new data for the improved and optimized use of FMT.

## Materials and Methods

### Animals

Eight to twelve weeks male C57BL/6 mice (weight ~25 g) were purchased from the experimental animal center of the second affiliated Hospital of Harbin Medical University and were acclimatized for 1 week before experiments were performed. They were reared in the Animal Laboratory Centre of Harbin Medical University under standard conditions (temperature 24–25°C, humidity 70–75%, with a 12 h light/dark lighting regimen) and were fed a standard diet of pellets and water *ad libitum*. The study was approved by HMU Medical Science Ethics Committee.

### Chemicals and Reagents

N-benzoyl-L-tyrosine ethyl ester (BTEE) and Nα-Benzoyl-L-arginine 4-nitroanilide hydrochloride (BAPNA) were purchased from Sigma-Aldrich (St. Louis, MO, USA). 4-Nitrophenyl β-D-glucopyranoside was purchased from BBI Life Sciences. DSS (MW: 36–50 kDa) was obtained from MP Biomedical (Solon, OH, USA). The antibodies used in this study were anti-TLR4 (19811-1-AP, Proteintech), anti-MyD88 (#4283, Cell Signaling Technology), anti-TRAF6 (YT4720, Immunoway), anti-inhibitor of NF-κB alpha (IκBα) (#4814, Cell Signaling Technology), anti-Occludin (13409-1-AP, Proteintech), anti-Claudin-1(13050-1-AP, Proteintech). Anti-GAPDH, goat anti-rabbit IgG and goat anti-mouse IgG were purchased from ZSGB-BIO Co. Ltd (Beijing, China). All other reagents used were of analytical grade.

### Preparation of FMT

Fresh fecal pellets from control mice were collected, added with sterile PBS at 10 mg/ml, homogenized, and divided into four preparations. The first one is the native fecal suspension without any further preparation; For the second preparation, fecal suspension was first centrifuged at 4,000 rpm for 10 min at 4°C, then the supernatant was taken and filtered through a disposable needle filter, and then collected (thus it only contains fecal supernatant); The third preparation is the re-suspension of the residual precipitate after centrifuge with equal volume of sterile PBS (thus it only contains fecal bacteria); While the fourth preparation is the boiling of an aliquot of the preparation 3 at 100°C for 30 min (thus it contains the killed fecal bacteria). The above four preparations were stored at −80°C until use.

### Induction of Colitis and Treatment With FMT

Colitis was induced by putting 3% DSS in drinking water for 7 days (Chassaing et al., 2014). The mice were divided into six groups: the control group with drinking water free of DSS, a colitis group treated with DSS but no fecal material, and another four groups treated with both DSS and the four FMT preparations described above, respectively. The fecal preparations were administrated to mice by intra-gastric gavage of 200 μl solution once a day for the first 2 days of DSS treatment (Yao et al., 2017). During the study, weight, physical condition, stool consistency, and the presence of occult blood in the feces were examined and documented daily. All animals were euthanized after 7 days of experiment by intraperitoneal injection of overdose amobarbital sodium. The entire colon (from the proximal colon to the anus), the total feces of mice were carefully removed, measured and weighted, then stored at −80°C for further analysis.

### Disease Activity Index

The disease activity index (DAI) was calculated for each animal with the added scores of body weight, stool consistency and stool blood as listed in the Supplementary Table 1 and described in our previous studies (Zhou et al., 2017).

### Histology Analysis and Scoring of Colonic Damage

Colonic tissues fixed in 4% (w/v) paraformaldehyde were paraffin-embedded and sliced into 5-μm sections, followed by staining with hematoxylin and eosin (HE) for light microscopic examination to assess colon injury and inflammation. Colonic damage was graded in a blinded manner as described in Supplementary Table 2 and in our other previous studies (Zhou et al., 2017).

### Determination of Fecal β-Glucuronidase, Trypsin, and Chymotrypsin Activity

β-glucuronidase, trypsin, and chymotrypsin (amidase) activities were measured by spectrophotometry using 4-Nitrophenyl β-D-glucopyranoside, Nα-Benzoyl-L-arginine 4-nitroanilide hydrochloride (BAPNA), and N-benzoyl-l-tyrosine ethyl ester (BTEE) as the substrate, respectively, with methods described in detail in studies by us and others (Dabek et al., 2008; Zhou et al., 2014).

### Colon RNA Extraction and qRT-PCR

Total RNA was isolated from colon tissues by using UNlQ-10 Column Trizol Total RNA Isolation Kit (Sangon Biotech, Shanghai, China). Then the RNA concentration and OD260/280 absorbance ratio were measured using a Nanodrop ND-1000 Spectrophotometer (Thermo Fisher Scientific, Waltham, MA, USA). The RNA was transcribed into cDNA using 5X All-In-One RT MasterMix (abm, Jiangsu, China; cat.no. G492) in Eppendorf Mastercycler® personal Thermo cycler at 42°C for 15 min, followed by 85°C for 5 min and then cooled at 4°C. Quantitative real-time polymerase chain reaction (qRT-PCR) was performed in volumes of 20 μl containing 1 μl of each primer (Supplementary Table 3) with the FastStart Universal SYBR Green Master (Roche, Basel, Switzerland; cat.no.04913850001) according to the manufacturer's instructions. PCR amplification was performed with the following conditions: 30 s at 95°C, 40 cycles followed by 5 s at 95°C and 31 s at 60°C. After that, a melting curve analysis was performed to confirm the specificity of the qRT-PCR. All samples were analyzed in triplicate, and the results were normalized to the expression of GAPDH. The results were calculated by the equation 2^−ΔΔCt^.

### Western Blot

Segments of colon were homogenized using RIPA buffer and protein inhibitor cocktail (1:10) (PhosSTOP ESAYpack, Roche). The homogenates were kept on ice for 30 min and centrifuged at 12,000 g for 5 min at 4°C. The protein concentration was determined using the BCA Protein Assay Kit (Beyotime, Shanghai, China). For western blot analysis, 20–80 μg proteins were electro-blotted onto a PVDF membrane following separation on 10% SDS-polyacrylamide gel electrophoresis. The immunoblot was then incubated with primary antibodies against Occludin, Claudin 1, TLR4, MyD88, TRAF6, and NF-κB inhibitor alpha (IκBα), or GAPDH. The chemiluminescence signals were analyzed using Quantity One (version 4.5.2) program (Bio-Rad Laboratories) and Image J software.

### Statistical Analysis

Results were expressed as mean ± SEM (*n* = 4–5 in each group). Differences between groups were determined by one-way ANOVA with Tukey's *post-hoc* test using Graphpad Prism version 5.0 (Graphpad Software, La Jolla, CA). Statistical significance was denoted with *P* < 0.05.

## Results

### Effects of Four Different Component of FMT on DSS-Induced Weight Loss, Disease, and Histological Scores in Mice

**Figure 1:**
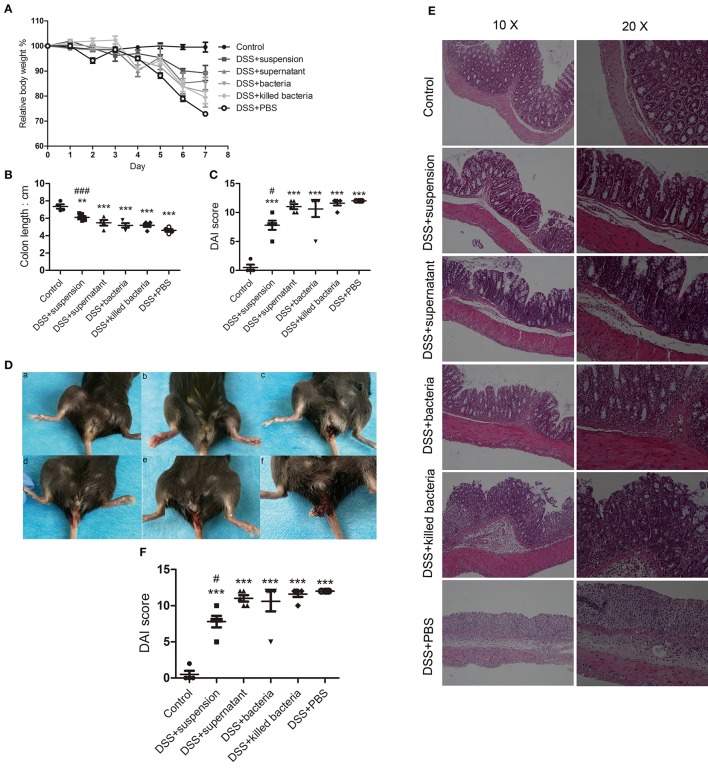
**(A)** Body weights, **(B)** colon length, **(C)** disease scores, **(D)** anal bleeding (a, Contol; b, DSS+suspension; c, DSS+supernatant; d, DSS+bacteria; e, DSS+killed bacteria; f, DSS+PBS), **(E)** histological stain, and **(F)** histological score. Data in **(A–C,F)** are shown as means ± SEM. ^**^P < 0.01, ^***^P < 0.001 compared with control group, ^#^*P* < 0.05, ^###^*P*< 0.001 compared with DSS+PBS group (one-way ANOVA with Tukey's *post-hoc* test).

The results showed that four different component of FMT all exerted some degrees of inhibition on DSS-induced colitis as exhibited by the less body weight loss, lower disease scores, and less shortening of colons, less anal bleeding, less inflammation manifestation, and less histological scores (Figures 1A–F), Among them, transplantation of fecal suspension showed the most potent efficacy on the relief of colitis.

### Effects of Four Different Component of FMT on Intestinal Barrier Integrity and Activities of Fecal Trypsin, Chymotrypsin, and β-Glucuronidase of DSS-Induced Colitis Mice

**Figure 2:**
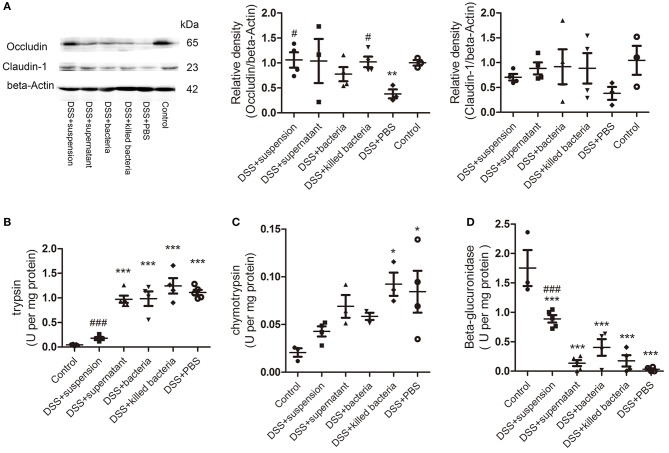
**(A)** The protein levels of occludin and claudin-1 in colon tissue, the relative intensity of occludin, and the relative intensity claudin-1. **(B)** Trypsin, **(C)** chymotrypsin, and **(D)** β-glucuronidase. Data in **(A–D)** are shown as means ± SEM. ^*^P < 0.05, ^**^P < 0.01, ^***^P < 0.001 compared with control group; ^#^*P* < 0.05, ^###^*P* < 0.001 compared with DSS+PBS group (one-way ANOVA with Tukey's *post-hoc* test).

To assess the integrity of the intestinal barrier, we measured the expression of tight junction proteins occludin and claudin-1 in colon tissue. We can see all four component of FMT increased both proteins, and transplantation of fecal suspension showed the most potent effect (Figure 2A). We proposed that β-glucuronidase played important role in intestinal barrier protection through catalyzing the formation of deconjugated bilirubin and further inactivating digestive proteases. To this regard, we assessed the activities of fecal β-glucuronidase and digestive proteases trypsin and chymotrypsin on day 7. The results indicated that transplantation of fecal suspension significantly decreased the digestive proteases trypsin and chymotrypsin along with increase in the activities of β-glucuronidase (Figures 2B–D).

### Effects of Four Different Component of FMT on Cytokines and Immune Cells of the Colon Tissue of DSS-Induced Colitis Mice

**Figure 3:**
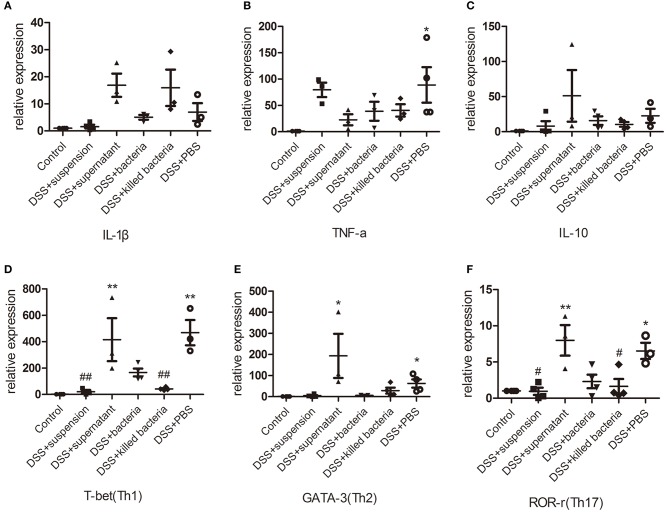
**(A–C)** The mRNA levels of cytokine, IL-1β, TNF-a, and IL-10, **(D–F)** the mRNA levels of immune cell, Th1, Th2, and Th17. Data in **(A–F)** are shown as means ± SEM. ^*^P < 0.05, ^**^P < 0.01 compared with control group; ^#^*P* < 0.05, ^##^*P* < 0.01 compared with DSS+PBS group (one-way ANOVA with Tukey's *post-hoc* test).

The mRNA levels of the pro-inflammatory cytokines, IL-1β and TNF-α, anti-inflammatory cytokine IL-10, immune cells, Th1, Th2, and Th17, in colon tissues of mice were determined. Results demonstrated that DSS-induced acute colitis was accompanied by significant increases in IL-1β, TNF-α, IL-10, Th1, Th2, and Th17 levels (Figures 3A–F); transplantation of fecal suspension restored these cytokines and immune cells back to normal levels except for TNF-α, while different components of FMT showed a different pattern of effects on these parameters (Figures 3A–F). It is worthy to mention that, transplantation of fecal bacteria showed certain mitigation effect that is similar but inferior to transplantation of fecal suspension (Figures 3A–F).

### Effects of Four Different Component of FMT on TLRs and TLR4-MyD88-TRAF6-NF-κB Signaling of DSS-Induced Colitis Mice

**Figure 4:**
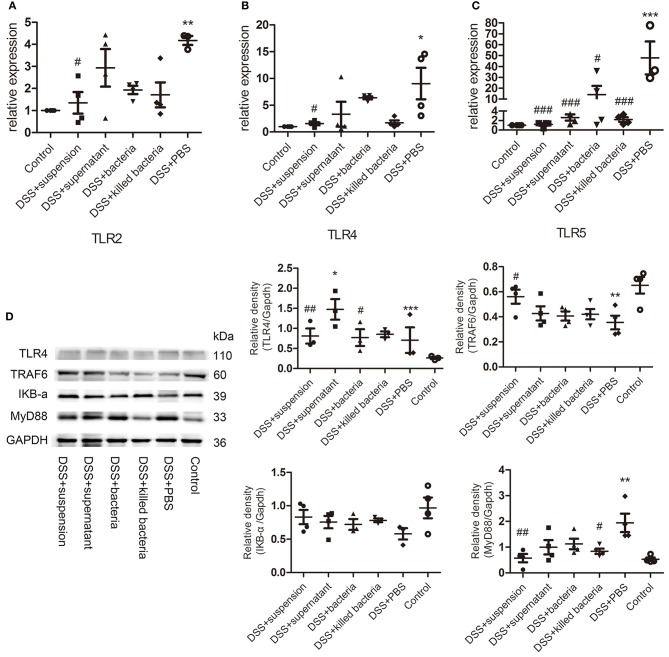
**(A–C)** The mRNA levels of TLRs, TLR2, TLR4, and TLR5, **(D)** the protein levels of TLR4, TRAF6, IKB-a, and MyD88 of colon tissue, the relative intensity of TLR4, TRAF6, IKB-a, and MyD88. Data in **(A–D)** are shown as means ± SEM. ^*^P < 0.05, ^**^P < 0.01, ^***^P < 0.001 compared with control group; ^#^*P* < 0.05, ^##^*P* < 0.01, ^###^*P* < 0.001 compared with DSS+PBS group (one-way ANOVA with Tukey's *post-hoc* test).

The mRNA levels of the TLRs, TLR2, TLR4, and TLR5 in colon tissues of mice were determined. The results suggested four different component of FMT reduced the expression of TLR2, TLR4, and TLR5 caused by DSS, and the transplantation of fecal suspension showed more potent effect than other three groups (Figures 4A–C). TLR4 can bind to pathogen-associated molecules, initiate intracellular signal transduction by medullary differentiation factor 88 (MyD88), and finally activate nuclear factor-κB (NF-κB), triggering a series of expression of inflammatory mediators and destroying the intestinal immune homeostasis eventually leads to the development of UC (Abreu et al., 2003; Bank et al., 2014; Choo et al., 2017). Therefore, we further examined the expression of key proteins in the TLR4-MyD88-TRAF6-NF-κB signaling pathway. We can see DSS caused remarkable increases in TLR 4 and MyD88, but decrease in TRAF6 and IκBα, while transplantation of suspension restored these changes toward normal (Figure 4D).

## Discussion

From the results above, we can see different degrees of protection by the four different components of FMT on the DSS-induced colitis, through improving the intestinal barrier integrity, reducing the cytokines and immune cells in colon and inhibiting the TLR4-MyD88-TRAF6-NF-κB signaling, accompanied by the remarkable reduction in digestive proteases trypsin and chymotrypsin and increase in β-glucuronidase. As DSS and FMT began at the same time, our results suggest that this intervention may have both prevention and treatment effects on colitis.

The standardized FMT is to obtain a highly purified flora in the laboratory by means of modern instrumentation, and then inject the quantified bacterial solution into the patient's intestine through an endoscope or a drainage tube (Zhang et al., 2013). However, our results suggested that transplantation of fecal suspension (contains both fecal bacteria and supernatant) showed better efficacy than transplantations of fecal supernatant, or live or killed fecal bacteria. Due to the fluorescence quenching effect of DSS, we failed to detect the fecal bacteria spectrum and mucosal spectrum, but our subsequent experimental results confirmed that transplantation of fecal suspension had better effect on improving the intestinal barrier integrity, reducing the cytokines, and immune cells in colon and inhibiting the TLR4-MyD88-TRAF6-NF-κB signaling pathway than other three component transplantation.

Increased intestinal mucosal permeability is considered to be a precursor to a variety of intestinal diseases, indicating the possible prerequisite of a dysfunction of intestinal mucosal barrier (Ménard et al., 2010). In recent years, lots of studies have suggested that the pathogenesis of UC and other autoimmune diseases are related to the increase of intestinal mucosal permeability and leaky gut (Hollander, 1999; Fasano, 2012; Sturniolo et al., 2012). Our results indicated that transplantation of fecal suspension had strong inhibition on digestive proteases trypsin and chymotrypsin but increased the activity of fecal β-glucuronidase, which is consistent with our previous study and our hypothesis about the pathogenesis of UC: the decrease of β-glucuronidase activity caused by intestinal flora disorder reduces the conversion of conjugated bilirubin to unconjugated bilirubin in the intestine, leading to impaired inactivation of digestive proteases and damage of intestinal mucosal barrier, increased infiltration of gut bacteria and bacterial toxins into the tissue and blood, which in turn induces a series of immune inflammatory reactions.

The therapeutic effect of FMT varies from person to person, with big differences in the colonization of new strains in the intestine after FMT (Angelberger et al., 2013). Our results showed that transplantation of fecal suspension (contains both fecal bacteria and supernatant) had superior alleviation of experimental colitis than transplantation of fecal bacteria only. Moreover, transplantation of fecal supernatant showed a certain mitigation effect, indicating that fecal supernatant may also contain effective components. Therefore, further experiments should be carried out to determine the specific substances and its mechanism. In summary, our research provide novel results that may help for a better understanding of the mechanism of FMT as well as improved and optimized application of FMT.

## Data Availability Statement

All datasets generated for this study are included in the article/Supplementary Material.

## Ethics Statement

This study has fully concerned relevant ethical principles and codes in the research design. The enactment of the study has strictly conformed to the plan approved by the ethics committee. Ethical requirements stated by Declaration of Helsinki and other international regulations are met by this study.

## Author Contributions

All authors participated in the design, interpretation of the studies and analysis of the data, and review of the manuscript. YH, XL, HY, and YG conducted the experiments. YH, XW and XQ wrote the manuscript. YL and MJ contributed to study supervision.

### Conflict of Interest

XQ is affiliated with GI Biopharma Inc. The remaining authors declare that the research was conducted in the absence of any commercial or financial relationships that could be construed as a potential conflict of interest.
