# Supplementary material for: The Functional Role of Fecal Microbiota Transplantation on Dextran Sulfate Sodium-Induced Colitis in Mice
Source: Front Cell Infect Microbiol. 2019 Nov 15;9:393. doi: 10.3389/fcimb.2019.00393 (PMC6873233; doi:10.3389/fcimb.2019.00393)
Supplement: Supplementary file 1 [file Table_1.docx]

**Supplementary Table 1.** The criteria of DAI

| DAI score | Weight loss(％) | Stool consistency | Occult/gross bleeding |
| --- | --- | --- | --- |
| 0 | None | Normal | Normal |
| 1 | 1-5 |  |  |
| 2 | 5-10 | Loose stools | Hemoccult positive |
| 3 | 10-15 |  |  |
| 4 | ＞15 | Diarrhea | Gross bleeding |

**Supplementary Table 2.** The criteria of histology analysis for colonic damage score

| Colon damage score | 0 | 1 | 2 | 3 |
| --- | --- | --- | --- | --- |
| Crypt architecture damage | None | Regeneration | Destruction |  |
| Edema in sub-mucosa | None | Mild | Moderate | Severe |
| Inflammatory cells infiltration | None /rare | Lamina propria | Sub-mucosa | Muscle layer |

**Supplementary Table 3.** Primers used for colon tissue RNA qRT-PCR

| Target gene | Primer sequence(5’-3’) |
| --- | --- |
| Gapdh | F: CATGGCCTTCCGTGTTCCTA |
|  | R: GCGGCACGTCAGATCCA |
| IL-1β | F: GAGCACCTTCTTTTCCTTCATCTT |
|  | R: TCACACACCAGCAGGTTATCATC |
| TNF-α | F: CATCTTCTCAAAATTCGAGTGACAA |
|  | R: TGGGAGTAGACAAGGTACAACCC |
| IL-10 | F: ATAACTGCACCCACTTCCCA |
|  | R: GGGCATCACTTCTACCAGGT |
| TLR2 | F: GAGTCTGCTGTGCCCTTCTC |
|  | R: GCTTTCTTGGGCTTCCTCTT |
| TLR4 | F: GCATGGCTTACACCACCTCT |
|  | R: GTCTCCACAGCCACCAGATT |
| TLR5 | F: CCACCGAAGACTGCGATGAAGAG  R: CCAGACCTTGTCCTTGAACACCAG |
| T-bet(Th1) | F：CCAGGGAACCGCTTATATGT  R：CTGGGTCACATTGTTGGAAG |
| GATA-3(Th2) | F：ACAGCTCTGGACTCTTCCCA  R：GTTCACACACTCCCTGCCTT |
| ROR-r(Th17) | F:CCACTGCATTCCCAGTTTCT  R:CGTAGAAGGTCCTCCAGTCG |
